# AI-integrated single-cell multi-omics decodes the hepatocellular carcinoma metabolism-immune axis: a new strategy for precision therapeutic targeting

**DOI:** 10.3389/fonc.2026.1915323

**Published:** 2026-08-05

**Authors:** Zihao Xu, Yifan Liu, Liangbin Cheng, Jun Xu

**Affiliations:** 1 School of Chinese Medicine, Hubei University of Chinese Medicine, Wuhan, Hubei, China; 2 Department of Hepatology, Hubei Provincial Hospital of Traditional Chinese Medicine Affiliated to Hubei University of Chinese Medicine, Wuhan, Hubei, China; 3 School of Basic Medical Sciences, Hubei University of Chinese Medicine, Wuhan, Hubei, China

**Keywords:** artificial intelligence, hepatocellular carcinoma, immune checkpoint inhibitor, metabolic checkpoint, single-cell multi-omics, tumor microenvironment

## Abstract

**Background:**

Immune checkpoint inhibitors have improved outcomes for hepatocellular carcinoma, yet most patients do not respond because the tumor’s metabolic environment suppresses immune cells. Single-cell RNA sequencing has revealed extensive immune diversity, but conventional analyses cannot link cell states to their physical location or to the metabolic signals that drive dysfunction.

**Methods:**

In this review, we examine how artificial intelligence, combined with single-cell and spatial multi-omics, can decode the metabolism-immunity network in liver cancer. We highlight two key metabolic switches: lipid uptake through a scavenger receptor that triggers ferroptosis in killer T cells, and lactate-induced changes in gene regulation that lock macrophages into a tumor-promoting state. We also summarize advanced computational tools including deep learning for data integration, spatial deconvolution, and foundation models that can infer metabolic activity from single-cell data and reconstruct cell movement over time.

**Results:**

These approaches enable researchers to identify key metabolic drivers of immune evasion and predict which checkpoints are most actionable.

**Conclusions:**

Artificial-intelligence-driven multi-omics transforms hepatocellular carcinoma research from descriptive catalogues into predictive, mechanism-based models, offering a roadmap for designing next-generation immunotherapies.

## 1 Introduction

Hepatocellular carcinoma (HCC) is the third leading cause of cancer-related mortality worldwide. Because most patients are diagnosed at intermediate or advanced stages, systemic therapy remains the absolute cornerstone for improving prognosis (1, 2). Immune checkpoint inhibitors (ICIs) have rewritten the first-line treatment landscape: the IMbrave150 trial established that atezolizumab plus bevacizumab significantly reduced the hazard ratio for overall survival (OS) to 0.58 (1), while the HIMALAYA trial demonstrated a positive OS hazard ratio of 0.78 with the STRIDE regimen (tremelimumab plus durvalumab) (2). Despite these advances, the clinical reality remains sobering: objective response rates persistently plateau at 20-30%, indicating that most patients harbor primary or acquired resistance to current immunotherapies. A root cause of this clinical impasse lies in the liver’s identity as the body’s central metabolic organ. The profoundly aberrant network of lipids, glucose, and amino acid metabolites within the hepatic tumor immune microenvironment (TIME) erects a formidable metabolic barrier that systematically remodels the functional state of tumor-infiltrating immune cells at both the epigenetic and signal-transduction levels.

Over the past decade, single-cell RNA sequencing (scRNA-seq) has evolved rapidly, revealing the cellular heterogeneity of the HCC TIME at unprecedented resolution. Early pioneering studies focused on constructing single-cell atlases: Zhang et al. built the first transcriptomic atlas of HCC immune cells in 2019, systematically delineating the subpopulation architecture of tumor-infiltrating lymphocytes and myeloid cells (3); Zheng et al. provided an in-depth portrait of the T cell landscape, quantifying for the first time at single-cell resolution the evolutionary trajectory of HCC-specific exhausted T cell subsets (4); subsequently, Xue et al. integrated nearly one million single cells from 124 patients and stratified the HCC TIME into five characteristic subtypes, each with a distinct immune composition and prognostic significance (5). Although these landmark studies established a solid foundation for HCC systems immunology, they remain fundamentally in a descriptive phase. They provide a detailed cell-type inventory but do not address the central mechanistic question: why these immune subsets fall into exhaustion or suppression in the first place.

Bridging from phenotypic description to causal mechanism requires overcoming three technical bottlenecks in current HCC multi-omics research. The first is the decoupling of spatial and metabolic gradients. Conventional single-cell dissociation strips away physical spatial coordinates, yet intra-tissue metabolite concentrations, such as those in hypoxic zones and high-lactate regions, exhibit pronounced spatial heterogeneity, severing the spatial evidence chain that links metabolism to immune state transitions. The second is the informational gap between molecular layers: transcript abundance (mRNA) does not directly equate to catalytic activity of the underlying metabolic enzymes or real-time metabolic flux. Inferring metabolic reprogramming solely from gene expression incurs systematic informational bias. The third is the limitation of linear analytical frameworks. Immune cell state evolution is a non-linear, dynamic process driven by coordination among multiple genes, multidimensional metabolites, and intercellular communication; conventional dimensionality reduction, clustering, and differential expression frameworks are inadequate for deciphering such high-dimensional causal regulatory networks.

It is against this backdrop that artificial intelligence (AI) and advanced computational science, particularly multimodal deep learning, graph neural networks (GNNs), and causal inference algorithms, have emerged as computational bridges to close these resolution gaps. By integrating multimodal data from single-cell transcriptomics, spatial transcriptomics, spatial proteomics, and metabolomics, cutting-edge AI models can reverse-infer metabolic activity at single-cell resolution and reconstruct the dynamic causal networks that govern immune cell fate decisions. This review aims to provide a systematic overview of this rapidly advancing interdisciplinary field. We first dissect the metabolic reprogramming landscape of the HCC immune microenvironment, then survey recent discoveries from single-cell and spatial omics technologies, followed by an in-depth analysis of innovations in AI-driven multimodal fusion frameworks, and finally re-examine HCC immune cell state trajectories through the lens of AI-integrated multi-omics and discuss the prospects for precision-targeted clinical translation.

A comprehensive literature search was conducted in PubMed, Embase, Web of Science, and IEEE Xplore (for artificial intelligence and computational methodologies) to identify relevant articles published between January 2020 and June 2026. Seminal studies published before 2020 were also included when they introduced foundational single-cell technologies (e.g., Monocle 3, Harmony, and RNA velocity), established landmark single-cell atlases of hepatocellular carcinoma (HCC), or defined key immunological concepts that continue to underpin the field. Search terms encompassed five major domains: (i) hepatocellular carcinoma and liver cancer; (ii) single-cell and spatial multi-omics (including scRNA-seq, scATAC-seq, spatial transcriptomics, spatial proteomics, and single-cell metabolomics); (iii) artificial intelligence and machine learning (including deep learning, graph neural networks, and foundation models); (iv) immune metabolism (including lipid, glucose, amino acid, and bile acid metabolism); and (v) cancer immunotherapy. Studies were eligible if they addressed at least two of these domains, were published in English, and appeared in peer-reviewed journals. Reference lists of all included articles were manually screened to identify additional relevant publications. As this article is a comprehensive narrative review, rather than a systematic review, a formal systematic-review reporting protocol based on the Preferred Reporting Items for Systematic Reviews and Meta-Analyses was not applied.

## 2 The metabolic reprogramming landscape of the HCC immune microenvironment

### 2.1 Aberrant lipid metabolism and immune suppression

Aberrant lipid metabolism is one of the most prominent features of the HCC microenvironment, and its impact on immune cell function represents the most thoroughly evidenced area of metabolism-immune crosstalk.

Within this complex network, excessive uptake of exogenous lipids and overproduction of endogenous lipids jointly drive immune cell functional collapse. On the exogenous uptake side, the scavenger receptor CD36 plays a central role. Ma et al. first established the “CD36-lipid-T cell ferroptosis” conceptual framework in 2021, demonstrating that excessive uptake of cholesterol and fatty acids by CD8+ T cells triggers lipid peroxidation and directly impairs their cytotoxic function (6). Qin et al. subsequently revealed a deeper layer of mechanism, showing that CD36 activates the p38-CEBPB-TfR1 signaling cascade to induce iron overload in T cells (7). This CD36-mediated immune suppression also exhibits etiology specificity: Leng et al. found that in metabolic dysfunction-associated steatohepatitis (MASH)-driven HCC, LIX1L specifically stabilizes CD36 protein expression, thereby accelerating tumor progression (8). Given CD36’s pivotal position in lipid metabolism targeting, Tzeng et al. developed PLT012, the first anti-CD36 IgG4 antibody, which successfully reshaped the TIME in preclinical models, demonstrating substantial translational potential (9).

Beyond exogenous uptake, dysregulated endogenous lipid synthesis and subsequent post-translational protein modifications constitute another core dimension governing immune exhaustion. Liang et al. identified a critical upstream regulatory circuit: Riplet (RNF135) deficiency leads to aberrant accumulation of fatty acid synthase (FASN) and massive palmitate production, which in turn drives CD8+ T cells into terminal exhaustion through STAT3 palmitoylation (10). This lipid-mediated protein S-acylation extends to the cholesterol metabolism branch: Wu et al. found that ZDHHC3-mediated SCAP S-palmitoylation promotes cholesterol synthesis and suppresses CD4+ T cell function, indicating that post-translational modifications represent a chronically underappreciated layer of lipid-immune regulation (11). Furthermore, Wen et al. demonstrated that squalene epoxidase (SQLE)-driven endogenous cholesterol accumulation severely damages mitochondrial function in CD8+ T cells, and that the SQLE inhibitor terbinafine effectively restores the efficacy of anti-PD-1 therapy (12).

The immune regulatory network shaped by lipid metabolism extends well beyond CD8+ T cell-intrinsic dysfunction; the remodeling of myeloid cells within the TIME constitutes an equally critical component of immune tolerance. In HCC, abnormal lipid accumulation in myeloid cells not only alters their intrinsic phenotype but amplifies immunosuppressive effects through complex intercellular crosstalk. Wang et al. showed that lipid droplet-laden tumor-associated macrophages (TAMs) secrete abundant CCL20, which specifically recruits CCR6+ regulatory T cells (Tregs) to construct a robust immunosuppressive microenvironment (13). The formation of this aberrant lipid phenotype is subject to even deeper post-transcriptional regulation: Wang et al. demonstrated that the splicing factor RBM17 reshapes lipid metabolism-related gene expression through an alternative splicing network, driving M2 macrophage polarization and suppressing CD8+ T cells, thereby formally incorporating RNA alternative splicing into the HCC metabolism-immune axis (14). Notably, lipid toxicity is broadly applicable to myeloid cells: dendritic cells (DCs) in the HCC microenvironment also suffer impaired cross-presentation due to excessive lipid accumulation, though this dimension still awaits systematic decoding at the multi-omics level.

When examining the lipid metabolic interplay between myeloid and lymphoid compartments, fatty acid oxidation (FAO) and its key shuttle proteins, the fatty acid-binding protein (FABP) family, exhibit pronounced cell-type specificity and functional duality. In TAMs, FABP-driven FAO displays well-documented pro-tumor effects: Tang et al. showed that FABP1 upregulates PPAR-gamma and CD36 to drive FAO in TAMs, and that the pan-FAO inhibitor orlistat produces significant synergy with anti-PD-1 therapy (15); similarly, Liu et al. found that FABP5-mediated FAO in TAMs not only promotes IL-10 production but also upregulates PD-L1 on Tregs (16). However, metabolic intervention is often a double-edged sword. The same study by Liu et al. noted that high FABP5 expression in exhausted CD8+ T cells (Tex) positively correlates with better survival status (16). Although Sun et al. confirmed in an obesity-associated HCC model that suppressing FABP5 activates CD8+ T cells through ferroptosis induction to exert antitumor effects (17), the opposing roles of the FABP family across different immune cell types raise an important caveat: systemic inhibition of FAO or lipid transport may inadvertently deprive Tex cells of the metabolic fitness required for sustained antitumor activity while reducing TAM-mediated immunosuppression.

### 2.2 Hypoxia and the glycolytic pathway

Hypoxia is a hallmark physical feature of the HCC microenvironment. Through stabilization of hypoxia-inducible factor 1-alpha (HIF-1alpha), tumor cell metabolism is forcibly reprogrammed toward aerobic glycolysis (the Warburg effect). Beyond the classical hypoxia-inducible pathway, protein ubiquitination also plays a critical upstream regulatory role in glycolytic remodeling. Chen et al. revealed the PSME3-FBXL7-PTEN axis: PSME3 promotes FBXL7-mediated PTEN degradation, markedly enhancing glycolytic activity in HCC cells and upregulating osteopontin secretion to drive Treg differentiation (18). This finding tightly links the ubiquitin-proteasome system, glucose metabolism, and immune suppression. Concurrently, HIF-1alpha also drives the CD39/CD73-adenosine pathway, which cooperates with glycolytic products to erect a robust immunosuppressive barrier (19).

Among the downstream consequences of glycolysis, lactate has undergone a fundamental re-evaluation, rising from its traditional status as a metabolic waste product to a central epigenetic messenger linking tumor and immune cells. Cai et al. established a complete “glycolysis-lactate-immune suppression” causal chain: the SRSF10-MYB signaling axis in HCC cells drives glycolytic gene expression, and the resulting L-lactate is exported via monocarboxylate transporters and taken up by TAMs, where it drives H3K18 histone lactylation (20). This epigenetic modification directly upregulates SRSF10 in TAMs, forming a unique trans-cellular positive feedback loop that stably maintains TAMs into a pro-tumor M2-polarized state. Subsequently, the same team identified NUPR1 as another key M2 polarization mediator governed by lactylation in 2025 (21), suggesting that the lactate-driven epigenetic remodeling network is considerably larger than initially appreciated.

Lactate-mediated immune suppression exhibits not only molecular depth but also pronounced etiology specificity and stereochemical specificity. In non-viral HCC, Yasukawa et al. discovered that loss of activin A receptor type 2A (ACVR2A) drives tumor cells into a hyperglycolytic state, and its MCT4-mediated massive lactate efflux specifically recruits Tregs (22). More intriguingly, the immune regulatory effects of lactate signaling depend on its stereoisomeric form. Han et al. found that gut microbiota-derived D-lactate reverses TAM polarization from M2 toward M1 via the PI3K/Akt pathway, exhibiting an antagonistic effect diametrically opposed to that of tumor-derived L-lactate (23). This competitive interaction between D-lactate and L-lactate not only reveals a refined interface of host-microbe interplay within the gut-liver axis but also provides a novel perspective for modulating the hepatic immune microenvironment through gut microecological intervention.

### 2.3 Amino acid and bile acid metabolism

The liver’s identity as the central hub for amino acid and bile acid synthesis endows the HCC microenvironment with uniquely organ-specific metabolic features. In this dimension, tumor cells construct a complex systemic immunosuppressive network not only by altering local metabolite concentrations but also through long-range interactions with the gut microbiota. Wei et al. in 2025 delineated a complete “liver-gut-liver” bile acid metabolic immune circuit: loss of AKR1D1 (steroid-5-beta-reductase) in HCC cells leads to aberrant expansion of *Bacteroides ovatus* in the gut, driving massive conversion of chenodeoxycholic acid into isolithocholic acid (iso-LCA); the enriched iso-LCA subsequently refluxes to the liver and directly impairs natural killer (NK) cell cytotoxic function by inhibiting p-CREB1 signaling, an effect reversible by spironolactone (24).

Beyond the distinctive bile acid pathway, amino acid imbalance and competition also act as key accelerators of immune exhaustion. SLC3A2-mediated lysine uptake enables tumor cells to directly compete with T cells for essential nutrients in the microenvironment, causing metabolic starvation of T cells, while exogenous lysine supplementation enhances the efficacy of anti-PD-1 combination therapy (25). In the classical tryptophan metabolism pathway, Wang et al. found that ZNF207 specifically upregulates IDO1, exacerbating tryptophan depletion and producing immunosuppressive kynurenine, further promoting CD8+ T-cell exhaustion at the transcriptional regulatory level (26).

This intense liver-specific metabolic stress exerts profound remodeling effects on the abundant innate-like T cell populations residing in the liver. Mucosal-associated invariant T (MAIT) cells and invariant natural killer T (iNKT) cells serve as bridges linking early metabolic sensing to immune defense, yet both succumb to metabolism-driven functional paralysis under the complex etiological backdrop of HCC. Cheng et al. discovered that long-chain acylcarnitines aberrantly accumulated in hepatitis B virus (HBV)-related HCC directly induce iNKT cell senescence (27); Deschler et al. revealed that under metabolic dysfunction-associated steatotic liver disease (MASLD) conditions, the lipotoxicity of polyunsaturated fatty acids (PUFAs) triggers MAIT cell ferroptosis (28). Surviving MAIT cells may even be co-opted by the microenvironment: Ruf et al. observed that TAMs at the tumor invasive margin specifically impair MAIT cell antitumor activity (29); Zhou et al. further delineated a “MAIT-TNF-TNFRSF1B-Treg” immunosuppressive signaling cascade, demonstrating that TNF secreted by MAIT cells paradoxically enhances Treg suppressive function (30). Moreover, innate immune cells display remarkable lineage plasticity under metabolic cues, as confirmed by Heinrich et al., who showed that NK cells can undergo irreversible phenotypic conversion toward ILC1 and subsequently toward pro-tumor ILC2 under the traction of tumor-derived cytokine gradients (31).

**Figure 1:**
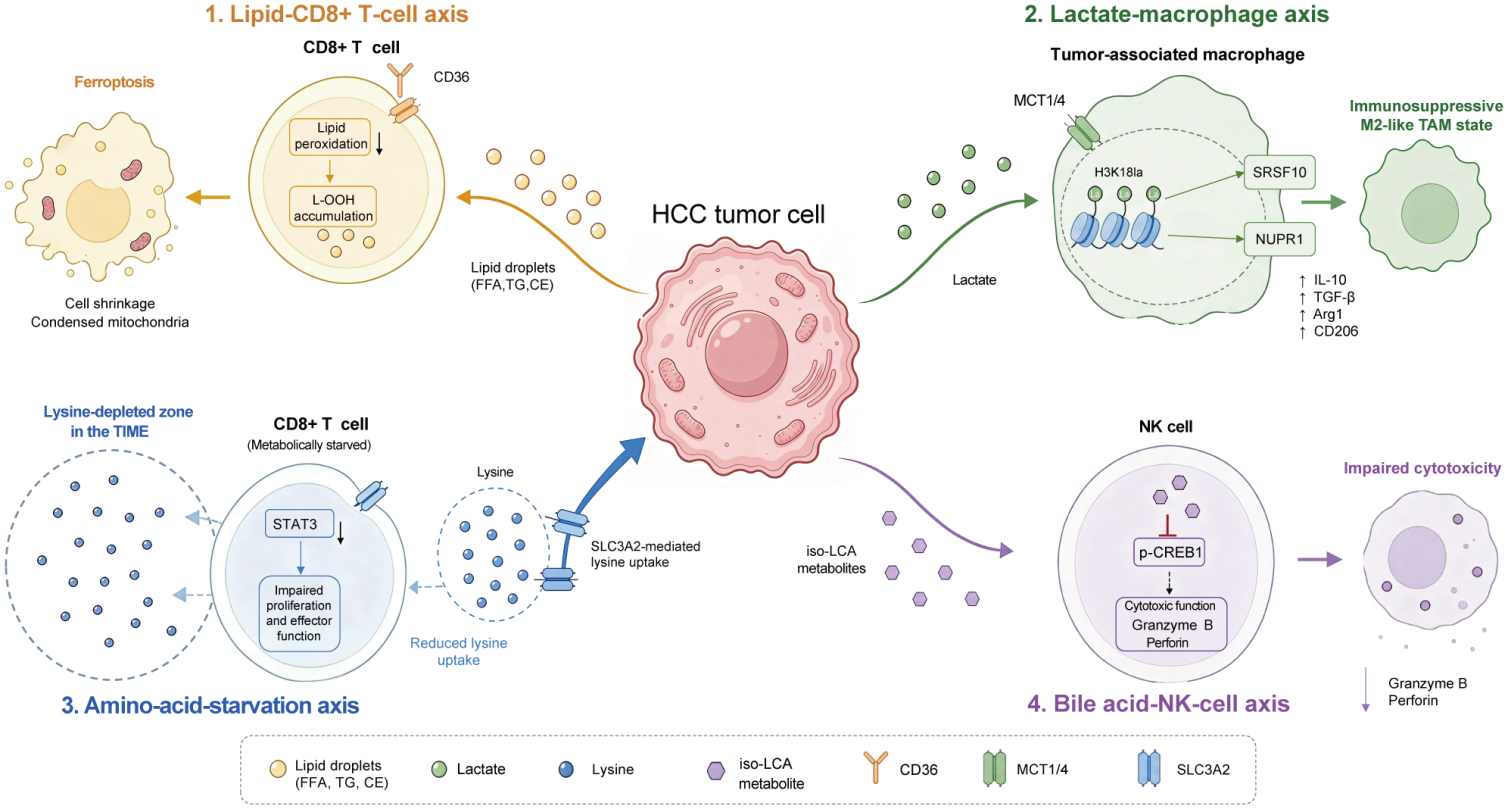
Major metabolism-immune axes in the tumor immune microenvironment of hepatocellular carcinoma (HCC). The central HCC tumor cell acts as a metabolic hub that contributes to immune dysfunction through four interconnected pathways. (1) Lipid-CD8+ T-cell axis: CD36-mediated lipid uptake promotes lipid peroxidation and lipid hydroperoxide (L-OOH) accumulation in CD8+ T cells, contributing to ferroptosis. (2) Lactate-macrophage axis: Tumor-derived lactate is transported through monocarboxylate transporters and taken up by tumor-associated macrophages (TAMs), where it induces histone H3 lysine 18 lactylation (H3K18la). H3K18la-associated SRSF10 and NUPR1 programs promote an immunosuppressive M2-like TAM state characterized by increased IL-10, TGF-beta, Arg1, and CD206. (3) Amino-acid-starvation axis: SLC3A2-mediated lysine uptake by HCC cells contributes to the formation of a lysine-depleted zone within the tumor immune microenvironment (TIME). Reduced lysine availability decreases STAT3 signaling in neighboring CD8+ T cells, thereby impairing their proliferation and effector function. (4) Bile acid-NK-cell axis: Tumor-associated bile acid metabolites, including isolithocholic acid (iso-LCA), suppress phosphorylated cAMP response element-binding protein 1 (p-CREB1) signaling in natural killer (NK) cells and reduce granzyme B and perforin expression, resulting in impaired cytotoxicity. Arrows summarize the principal directions of the mechanisms discussed in the main text.

Such a highly complex, spatially biased metabolic-innate immune interaction network, characterized by invasive-margin intercellular communication and concentration gradients of microbiota-derived molecules, can no longer be comprehensively resolved by conventional flow cytometry alone. The introduction of spatial multi-omics technologies has provided powerful tools to break this impasse. For example, recent studies employing spatial proteomics have precisely localized the spatial co-localization of specific F5 cancer-associated fibroblasts (CAFs) with cancer stem cells, confirming that stromal cells not only provide a physical scaffold but are central participants in shaping the local metabolic-immune microenvironment (32). The core molecular mechanisms of the HCC metabolism-immune axis are summarized in **Figure 1**.

## 3 Frontiers in single-cell multi-omics and spatial omics technologies

### 3.1 Single-cell transcriptomics and epigenomics

The widespread adoption of scRNA-seq and single-cell ATAC sequencing (scATAC-seq) has driven HCC immune microenvironment research from macroscopic phenotypic description toward lineage tracing at single-cell resolution. In foundational atlas construction, the early pioneering work of Zheng et al. systematically delineated the functional spectrum of T cells in HCC, quantifying for the first time the gradient trajectory of CD8+ T cells toward exhaustion and revealing the pronounced heterogeneity of Tregs (4). Subsequently, Zhang et al. constructed the first single-cell atlas covering the full spectrum of HCC immune cells, precisely defining the polarization continuum of TAMs and dendritic cell (DC) subsets (3).

As single-cell cohort sizes expanded, the clinical stratification and prognostic value of these atlases became increasingly evident. Xue et al., through large-scale integrative analysis, stratified the TIME of 124 patients into five characteristic subtypes, each possessing a distinct immune cell composition and independent survival outcome (5). Sun et al. demonstrated that enrichment of the KLRB1+ CD8+ T cell subset within the microenvironment is closely associated with early HCC recurrence (33). Delving into specific etiologies and disease stages, Ho et al. identified a TIGIT-NECTIN2 immunosuppressive axis unique to HBV-associated HCC, substantially enriching the dimension of etiology-specific immune regulation (34). Lu et al., by comparing primary and metastatic lesions, found that PPAR-gamma-driven MMP9+ TAMs undergo specific expansion in the metastatic microenvironment (35).

The spatiotemporal heterogeneity of gene expression is fundamentally governed by epigenetic network remodeling, and scATAC-seq technology provides a critical entry point for decoding this regulatory logic. You et al. discovered that in HBV-specific CD8+ T cells, chromatin accessibility at binding sites of key transcription factors such as NFKB1/2 and NFATC2 is significantly altered, and these epigenetic remodeling events are closely linked to terminal T cell exhaustion (36). Craig et al., through cross-cancer deep epigenomic comparison, extracted 31 core transcription factor regulon activity signatures, successfully achieving accurate discrimination between HCC and intrahepatic cholangiocarcinoma (iCCA) (37). Furthermore, by combining single-cell-level ligand-receptor (L-R) inference, Song et al. precisely identified the specific chemokine interaction pair between CCL18+ M2 macrophages and XCL1+ CD8+ T cells, providing high-dimensional molecular evidence for understanding intercellular communication underlying complex phenotypes (38).

### 3.2 Spatial multi-omics atlases: reconstructing the physical topological interface of metabolism and immunity

Although scRNA-seq resolves the cellular composition of the microenvironment at unprecedented resolution, it completely loses the physical spatial coordinates of cells during tissue dissociation. Yet hypoxia-driven metabolite concentration gradients and intercellular communication interactions are both fundamentally dependent on spatial physical topology. Across multiple independent spatial omics studies spanning 2023 to 2025, investigators have consistently identified the tumor-stroma boundary as the most dynamically active physical interface for metabolism-immune crosstalk.

In revealing the heterogeneity of the spatial invasive front, Wu et al. employed spatial transcriptomics to precisely localize an invasive zone approximately 500 um in width, finding that serum amyloid A-mediated macrophage chemotaxis and M2 polarization are highly active within this physical region (39). Although the exact topological width of this invasive zone may vary with HCC etiology and tumor stage, the concept that the peri-tumoral interface is the critical battleground for metabolism-immune interplay has been widely corroborated. Qiu et al., using multiplexed immunofluorescence, demonstrated that VIM-high macrophage subpopulations at the tumor margin specifically recruit Tregs through IL-1beta secretion (40). Yang et al. identified PD-L1+ CD103+ DCs enriched in peri-tumoral regions as independent spatial markers predicting early HCC recurrence (41). Zhang et al. found that in the neoadjuvant therapy setting, B cell expansion and PAX5 signature gene expression are spatially enriched in tertiary lymphoid structure (TLS) regions (42).

Spatial omics further extends the analytical dimension to microscopic distances and the spatial niches of cell subpopulations. Sheng et al., using imaging mass cytometry, revealed that tissue-resident Kupffer cells and infiltrating macrophages occupy entirely distinct spatial territories in HCC and execute functionally opposing immunoregulatory roles (43). Maestri et al. provided direct evidence from a geometric topology perspective that the absolute physical distance between CD8+ T cells and tumor cells carries independent prognostic stratification value (44). Such three-dimensional cell interactions are not only constrained by physical distance but also follow specific molecular mechanisms. Ma et al., combining multi-region scRNA-seq, discovered that tumor cell biodiversity reshapes the TIME through secretion of specific signaling factors (45), and that ligand-receptor interactions such as LGALS9-SLC1A5 and SPP1-PTGER4 follow precise lock-and-key mechanisms to spatially organize the physical recruitment and colonization of specific immune cells (46).

## 4 AI and computational frameworks: engines for multidimensional data fusion

**Figure 2:**
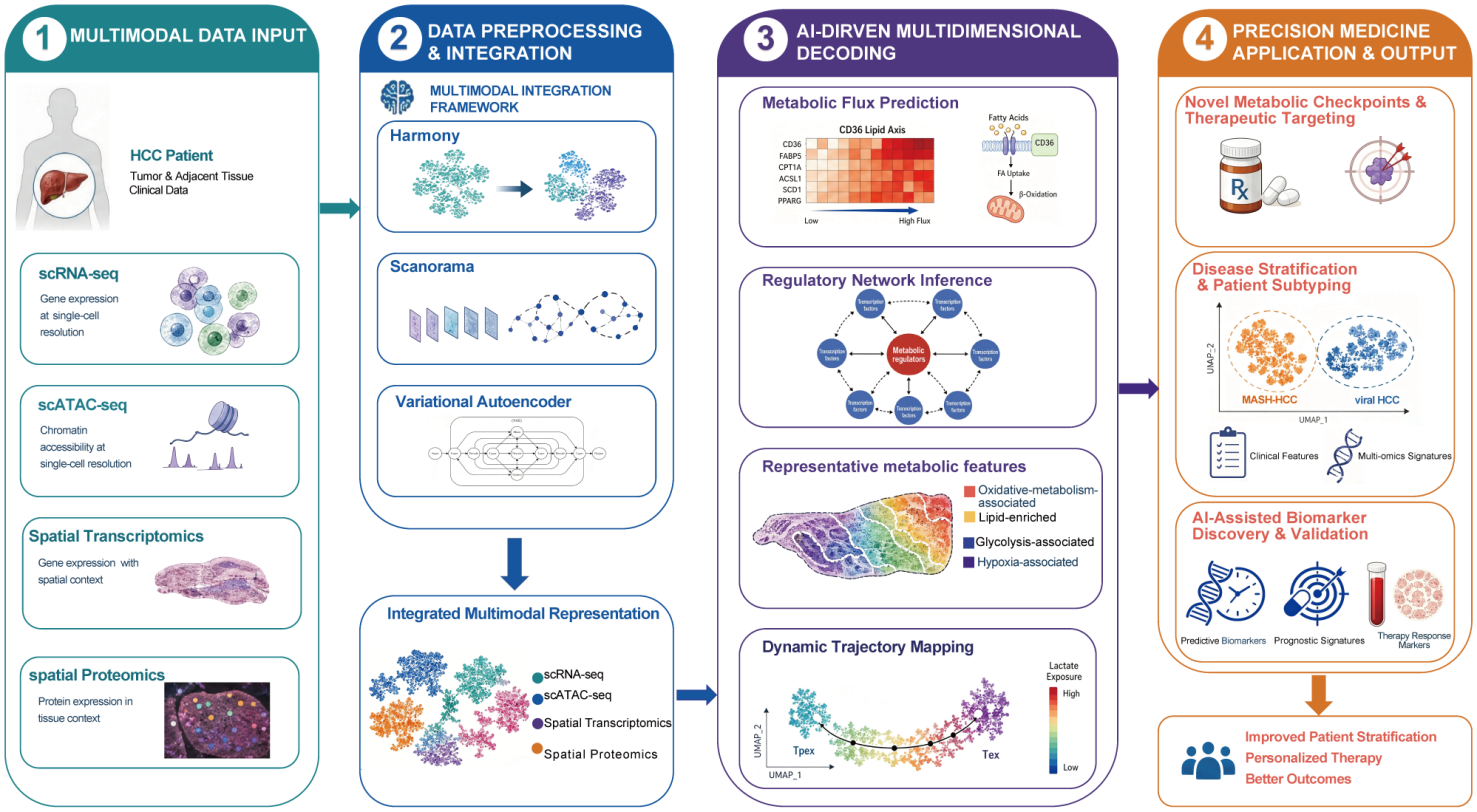
Conceptual workflow for multimodal single-cell and spatial-omics analysis of the hepatocellular carcinoma (HCC) microenvironment. The proposed workflow comprises four stages. (1) Multimodal data input: single-cell RNA sequencing (scRNA-seq), single-cell assay for transposase-accessible chromatin using sequencing (scATAC-seq), spatial transcriptomics, spatial proteomics, and associated clinical information are collected from HCC tumor and adjacent tissues. (2) Data preprocessing and integration: batch-correction and integration methods, such as Harmony and Scanorama, together with variational autoencoders, generate an integrated multimodal representation. (3) Artificial intelligence (AI)-driven multidimensional decoding: computational approaches can support metabolic-flux prediction, gene regulatory network inference, identification of representative spatial metabolic features, and dynamic trajectory mapping from progenitor exhausted T cells (Tpex) toward terminally exhausted T cells (Tex). (4) Precision-medicine applications and outputs: integrated analyses may facilitate the nomination of metabolic checkpoints, disease stratification and patient subtyping, and AI-assisted biomarker discovery and validation, ultimately supporting patient stratification and personalized therapeutic development. All plots and analytical outputs shown are conceptual illustrations of the reviewed workflow and do not represent original patient-level analyses performed in this review.

Faced with fundamental differences in resolution, data sparsity, and measurement principles across multimodal omics data, AI and advanced computational frameworks have become essential for closing the resolution gap and converting massive data into biological insight. This section systematically reviews the application paradigms and frontier developments of these frameworks in HCC metabolism-immune research across five interlocking computational dimensions. The AI-driven single-cell multi-omics integration and analysis workflow is illustrated in **Figure 2**.

### 4.1 Data integration and batch correction

When integrating data from different sequencing platforms, multi-center laboratories, and heterogeneous HCC patient cohorts, eliminating technical batch effects is the cornerstone for establishing a unified analytical framework. Early linear correction methods have been supplanted by more scalable machine learning algorithms. Harmony uses iterative clustering with soft cluster center correction to efficiently project heterogeneous data into a shared low-dimensional embedding space, offering substantial computational advantages for processing million-cell atlases (47). Scanorama employs mutual nearest neighbors to accurately identify matching cell anchors across batches and perform panoramic stitching, effectively removing technical offsets while maximally preserving the intrinsic biological heterogeneity of HCC (48). LIGER innovatively adopts integrative non-negative matrix factorization (iNMF), extracting shared and dataset-specific meta-features to provide a matrix decomposition pathway distinct from deep learning approaches (49). In multimodal alignment, Seurat v4 introduced the weighted nearest neighbor (WNN) algorithm, which breaks through single-modality limitations and provides a unified mathematical framework for the joint clustering of RNA, ATAC, and surface protein data from the same single cell, and has become an industry benchmark for defining complex immune subsets (50).

### 4.2 Metabolic flux prediction and cross-modal deep learning integration

Given the current technical bottlenecks of high-throughput single-cell metabolomics, using AI to reverse-infer single-cell metabolic activity from transcriptomic data has become the key computational bridge for resolving metabolic reprogramming in the TIME. The Compass algorithm innovatively couples flux balance analysis (FBA) with the human global metabolic network reconstruction (Recon2), achieving the first quantitative estimation of single-cell metabolic flux from scRNA-seq data (51). To overcome noise interference from extreme sparsity in single-cell data, scFEA introduced a probabilistic hypergraph model with GNNs, substantially improving the robustness of metabolic flux estimation (52). Initial applications of these tools in the HCC immune microenvironment consistently anchor lipid metabolism and glycolysis as the most prominent metabolic axes driving immune cell state transitions.

In deep multimodal data integration, variational autoencoder (VAE)-based deep learning networks constitute the current core algorithmic foundation. scVI uses a VAE to achieve efficient batch correction and probabilistic generation; its advanced model scANVI supports semi-supervised cell-type annotation; and totalVI further integrates protein abundance into the latent space representation (53). To manage the computational burden of incremental data, scArches introduces network surgery and transfer learning strategies, allowing newly sequenced HCC samples to be directly projected onto pre-built reference atlases without costly model retraining (54). MultiVI achieves unified VAE modeling across RNA, ATAC, and protein modalities, marking the evolution of integration frameworks from bimodal to fully multimodal (55). For data silos lacking multimodally paired measurements, scJoint performs cross-modal label transfer through semi-supervised transfer learning (56), while UINMF specifically addresses the non-overlapping feature set challenge, allowing retention of dataset-specific features while sharing partial dimensional information (57).

In recent years, generative pretrained transformer models have sparked a paradigm shift in systems biology. The foundation model scGPT, pretrained on over 33 million single-cell transcriptomes, demonstrates exceptional representation learning capability through HCC-specific fine-tuning (58). Geneformer, also built on a transformer architecture, offers the breakthrough capability of supporting *in silico* perturbation: accurately predicting the global impact of knocking out or overexpressing specific metabolic enzymes or immune checkpoints on HCC cellular network states without physical experimental intervention (59). Two recent studies have provided a more comprehensive picture of this rapidly evolving field: a systematic benchmark of six foundation models across gene-level and cell-level tasks found that no single model is universally superior and that simpler models can be more efficient under resource-constrained settings (60), while a critical evaluation of five foundation models for gene perturbation prediction demonstrated that none outperformed simple linear baselines on unseen perturbations, indicating that the capacity of current models to generalize beyond their training distribution remains limited (61).

### 4.3 Trajectory inference and cell fate prediction

The transition of immune cells from a highly cytotoxic effector state to terminal exhaustion is fundamentally a continuous spatiotemporal process driven by local metabolic gradients. Monocle 3 reconstructs pseudotime trajectories of cell differentiation by combining uniform manifold approximation and projection nonlinear dimensionality reduction with approximate graph abstraction (62); CytoTRACE takes a distinct approach, using transcriptional diversity entropy to quantify the undifferentiated potential of cells (63). To overcome the temporal limitation of static snapshot data, RNA velocity analysis introduced the ratio dynamics of unspliced to spliced mRNA, mathematically deriving the instantaneous direction of transcriptional dynamics (64); scVelo subsequently constructed a comprehensive nonlinear dynamical latent model, substantially improving the precision of velocity vector estimation (65). Building on this, CellRank deeply couples the RNA velocity field with a Markov chain model, enabling accurate quantification of the probability of cells flowing toward different terminal fates (66). In HCC research, this suite of dynamical models has been successfully applied to finely resolve the exhaustion trajectories of CD8+ T cells under the dual stress of persistent antigen exposure and metabolic perturbation.

### 4.4 AI integration of spatial transcriptomics

Seamlessly mapping high-dimensional single-cell transcriptomes onto spatial sections that preserve physical coordinates represents the core challenge for decoding HCC metabolic-immune spatial niches. Graph convolutional networks (GCNs) have demonstrated clear advantages in this arena. SpaGCN jointly encodes the gene expression matrix, spatial geometric coordinates, and visual features from H&E histological images in three dimensions, successfully identifying spatial domains with high biological coherence (67). GraphST employs a self-supervised contrastive learning strategy to enhance GNN feature extraction efficiency and is compatible with both spatial clustering and cell deconvolution tasks (68). STAGATE introduces an adaptive graph attention mechanism that dynamically adjusts the weights of neighboring nodes, thereby capturing complex tissue structural boundaries, such as the invasive front, with greater sensitivity (69).

For spatial deconvolution, Tangram uses a deep learning optimization algorithm to achieve high-precision alignment of single cells with spatial transcriptomic pixels (70); cell2location employs a Bayesian hierarchical model to quantify the spatial distribution abundance of rare immune subpopulations at sub-cellular resolution with unprecedented precision (71). DestVI further uses variational inference to estimate the continuous state gradients of cells along spatial physical distances, precisely capturing the micro-state differences of the same immune cell type under different metabolic gradients (72). Notably, Ye et al. recently achieved four-dimensional modal integration of scRNA-seq, spatial transcriptomics, spatial proteomics, and spatial metabolomics in HCC research, mathematically defining the specific spatial niches that determine early HCC recurrence, thereby establishing a proof-of-concept benchmark for multimodal spatial AI applications (73).

### 4.5 Gene regulatory network and ligand-receptor inference

Bridging from correlational description to causal inference is the ultimate goal of computational systems biology. In resolving cell-intrinsic driving forces, pySCENIC integrates transcription factor motif enrichment analysis with co-expression module networks, enabling high-throughput inference of regulon cis-regulatory activity at the scale of hundreds of thousands of single cells (74). CellOracle constructs quantitative gene regulatory networks (GRNs) and introduces an in silico transcription factor perturbation model to simulate the direction and signal amplitude of cell state transitions (75).

For decoding cell-extrinsic microenvironmental interactions, L-R communication network analysis provides the core perspective. NicheNet innovatively fuses prior signaling cascade networks with gene regulatory databases to reverse-infer the actual regulatory effects of specific microenvironmental ligand molecules on downstream gene expression programs in target cells (76). CellChat, based on a large-scale, manually curated signaling interaction database, quantitatively quantifies and compares the topological patterns and communication intensities of information flow among different immune subpopulations (77), providing a powerful computational tool for dissecting the complex metabolite-cytokine interaction networks within the HCC microenvironment.

Several critical limitations of current AI tools in HCC metabolism-immune research warrant acknowledgment: (1) no AI tool has undergone systematic benchmarking on HCC-specific datasets; (2) the flux balance analysis adopted by Compass assumes metabolic steady state, a condition that may be violated in activated immune cells undergoing rapid metabolic reprogramming; more fundamentally, these methods infer metabolic flux from mRNA abundance, which serves as a computational surrogate rather than a direct measurement of metabolite concentrations or enzymatic activity. Because mRNA levels are imperfect proxies for protein abundance, they cannot capture the post-translational regulation, allosteric modulation, and substrate availability that collectively determine real-time metabolic flux; (3) deep learning models such as scVI and scGPT are essentially black boxes, and their latent space representations are difficult to directly map to specific metabolic pathways or regulatory factors, limiting their utility in mechanism-oriented studies; (4) foundation models such as scGPT and Geneformer require graphics processing unit cluster support, with limited accessibility for routine HCC research laboratories; (5) foundation models lack HCC-specific fine-tuning validation, and their predictive reliability in the HCC microenvironment remains to be established. More broadly, a recent benchmark found that even for the well-defined task of gene perturbation effect prediction, existing foundation models, including scGPT, Geneformer, and scFoundation, do not outperform simple additive linear models when evaluated on perturbations not represented in the training data (78). Recent advances in agentic AI frameworks suggest a complementary strategy for addressing several of the pipeline standardization challenges discussed above. Workflow orchestration platforms such as LangChain, AutoGen, and CrewAI can, in principle, coordinate the sequential execution of specialized computational tools, including scVI for data integration, Compass for metabolic flux inference, Harmony for batch correction, and spatial deconvolution algorithms, within a unified decision-making framework that automates tool selection, parameter optimization, and provenance documentation. A recent systematic benchmark across eight large language models and three agent frameworks, namely ReAct, LangGraph, and AutoGen, for 50 single-cell omics tasks demonstrated that multi-agent architectures improve task completion quality and that self-reflection modules contribute more to performance than retrieval-augmented generation or planning (79). In a complementary demonstration, CellAtria, a LangGraph-based agentic framework, reduced scRNA-seq data ingestion and standardization from approximately 15 hours of manual bioinformatician effort to less than 10 minutes by using a large language model to orchestrate pre-vetted analytical tools (80). By encoding domain expertise as structured agent prompts and tool-use policies, such workflows could, in principle, reduce operator-dependent analytical variability and enforce consistent quality control thresholds across heterogeneous HCC patient cohorts. We emphasize, however, that no agentic workflow has been specifically validated for HCC multi-omics analysis. All demonstrations to date have been conducted in pan-cancer or non-cancer settings, and the applicability of these frameworks to HCC-specific challenges, including etiology-dependent metabolic heterogeneity and liver-specific immune compartment deconvolution, remains an open question. To facilitate reference and application by researchers in the field, the major AI computational frameworks reviewed above, their core algorithms, and their specific applicability and code access for HCC multi-omics analysis are systematically summarized in **Table 1**.

**Table 1:** Summary of major AI tools reviewed in this article.

Tool	Function category	Core algorithm	Input modality	Interpretability	HCC applicability, validation & limitations	Language	Ref.
scVI/scANVI/totalVI	Single-cell multimodal integration	Variational autoencoder (VAE)	scRNA-seq; totalVI additionally accepts CITE-seq protein data	Low	Pan-cancer general; directly usable. No HCC-specific benchmarking	Python (scvi-tools)	(53)
Harmony	Batch effect correction	Iterative soft k-means clustering correction in PCA space	scRNA-seq (operates on low-dimensional PCA embedding)	Medium	Pan-cancer general; computationally efficient at million-cell scale. Applied in HCC TIME subtyping by Xue et al.	R/Python	(47)
Scanorama	Heterogeneous data integration	Mutual nearest neighbors (MNN) with panoramic stitching	scRNA-seq	Medium	Pan-cancer general; designed for multi-center cohort stitching. No HCC-specific validation	Python	(48)
LIGER/UINMF	Multimodal data integration	Integrative non-negative matrix factorization (iNMF)	scRNA-seq/scATAC-seq/DNA methylation/spatial	Medium	Pan-cancer general; UINMF supports non-overlapping feature sets. No HCC-specific benchmarking	R (rliger)/GitHub	(49, 57)
Seurat v4 WNN	Multimodal joint clustering	Weighted nearest neighbor graph	scRNA-seq + scATAC-seq or CITE-seq; requires multimodal data from the same single cell	Medium	Pan-cancer general; directly usable. Applied in HCC studies. Requires CITE-seq or 10x Multiome paired measurements	R (Seurat v4+)	(50)
Compass	Metabolic flux reverse inference	Flux balance analysis (FBA) + Recon2/Recon3D global human metabolic network	scRNA-seq	Medium	Requires parameter tuning with HCC-specific metabolic model. Assumes metabolic steady state. Flux scores are computationally inferred surrogates, not direct metabolite measurements	Python (GitHub)	(51)
scFEA	Metabolic flux reverse inference	Probabilistic hypergraph model + graph neural network (GNN)	scRNA-seq	Low	Pan-cancer general; robust against sparse single-cell data noise. No HCC-specific validation	Python (GitHub)	(52)
scVelo/CellRank	Cell fate and trajectory inference	RNA velocity field with likelihood-based dynamical modeling + Markov chain	scRNA-seq; requires spliced and unspliced read counts	Medium	Pan-cancer general; resolves T cell exhaustion trajectories. Applied in HCC CD8+ T cell studies. Requires sufficient intronic read coverage	Python	(65, 66)
Monocle 3/CytoTRACE	Pseudotime trajectory inference	Approximate graph abstraction via UMAP/transcriptional diversity entropy	scRNA-seq	Medium	Pan-cancer general; directly usable. Applied in HCC single-cell studies	R (Monocle 3)/R (CytoTRACE)	(62, 63)
CellOracle	Gene regulatory network inference with in silico perturbation	GRN construction + in silico TF perturbation simulation	scRNA-seq + scATAC-seq; paired data required for GRN construction	Medium	Requires HCC-specific GRN with matched scATAC-seq TF motif data. No HCC-specific application reported	Python (celloracle)	(75)
SCENIC/pySCENIC	Regulon inference	GRNBoost2 co-expression + cisTarget motif enrichment + AUCell activity scoring	scRNA-seq	Medium	Pan-cancer general; directly usable. Applied to HCC by Craig et al. to discriminate HCC from iCCA using 31 regulon signatures	R/Python (pySCENIC)	(74)
NicheNet	Ligand-target gene effect inference	Integration of prior signaling pathway databases with GRNs; modified PageRank	scRNA-seq (sender and receiver cell expression)	Medium	Pan-cancer general; directly usable. Prior signaling networks not HCC-specific	R (nichenetr)	(76)
CellChat	Intercellular communication network inference	Manually curated L-R database (2,021 interactions, 229 pathways) + mass action model + graph theory	scRNA-seq	High	Pan-cancer general; directly usable. L-R interactions manually curated with literature support. No HCC-specific validation	R (CellChat)	(77)
SpaGCN/GraphST	Spatial domain identification	Graph convolutional network with joint encoding of expression, spatial coordinates, and H&E histology/self-supervised contrastive learning	Spatial transcriptomics; SpaGCN additionally accepts H&E histology images	Medium	Pan-cancer general. Applied in HCC spatial transcriptomic studies. GraphST supports both clustering and deconvolution	Python (GitHub)	(67, 68)
Tangram/cell2location	Spatial omics deconvolution	Deep learning optimization (PyTorch)/Bayesian hierarchical negative binomial model	scRNA-seq reference + spatial transcriptomics data	Medium	Pan-cancer general; resolves rare immune subpopulations at sub-cellular resolution. Applied in HCC spatial studies. cell2location provides uncertainty estimates	Python (GitHub)	(70, 71)
scGPT/Geneformer	Single-cell foundation model	Generative pretrained Transformer, 33M cells/encoder-only Transformer with ranked-gene tokenization, 30M cells	scRNA-seq; scGPT additionally supports scATAC-seq and CITE-seq	Low	Urgently requires HCC-specific fine-tuning. In silico perturbation prediction not yet superior to linear baselines in systematic benchmarks. GPU cluster support required	Python (GitHub)	(58, 59)

HCC, hepatocellular carcinoma; TIME, tumor immune microenvironment; scRNA-seq, single-cell RNA sequencing; scATAC-seq, single-cell assay for transposase-accessible chromatin using sequencing; VAE, variational autoencoder; MNN, mutual nearest neighbors; PCA, principal component analysis; WNN, weighted nearest neighbor; UMAP, uniform manifold approximation and projection; FBA, flux balance analysis; GNN, graph neural network; GRN, gene regulatory network; GCN, graph convolutional network; iNMF, integrative non-negative matrix factorization; CITE-seq, cellular indexing of transcriptomes and epitopes by sequencing; L-R, ligand-receptor; TF, transcription factor; GPU, graphics processing unit. Software names standardized per published documentation. Interpretability: High = transparent statistical model with direct biological mapping; Medium = partially interpretable with feature attribution possible; Low = deep learning model resistant to direct biological interpretation.

## 5 HCC immune cell state remodeling pathways through the lens of AI multi-omics

### 5.1 Metabolic regulation and functional exhaustion of T cell communities

When the multimodal AI frameworks described above are applied to HCC atlas analysis, researchers can reconstruct the full metabolic landscape of T cell state evolution at single-cell resolution. Computational trajectory inference reveals that the progression of CD8+ T cells from an effector state to Tex is not a discrete fate jump but a gradual process continuously shaped by the metabolic microenvironment.

Building on this insight, the concept of the metabolic checkpoint provides a core theoretical framework for understanding this spatiotemporal evolution. Just as PD-1 and CTLA-4 constrain immune signaling at the receptor level, specific metabolic enzymes and transporters gate the metabolic fitness of T cells at the level of metabolic flux. In the lipid metabolism axis, CD36, as the most representative metabolic checkpoint, not only mediates aberrant lipid uptake but also initiates the lethal “lipid accumulation-lipid peroxidation-ferroptosis” cascade (6). In endogenous synthesis, FASN drives terminal exhaustion through STAT3 palmitoylation (10), while SQLE-mediated cholesterol accumulation directly destroys the mitochondrial network (12). These three molecular pathways converge to reveal the severe metabolic dysfunction that CD8+ T cells suffer in the lipid-rich HCC microenvironment. Consistent with the updated hierarchical differentiation model, TCF1+PD-1int progenitor exhausted T cells (Tpex), rather than terminally exhausted Tex, are the core subset that responds to ICI therapy (81). This paradigm profoundly reorients the clinical logic of metabolic intervention: the true value of targeting CD36 or FASN may lie in preserving the metabolic homeostasis and differentiation potential of the Tpex cell pool, rather than futilely attempting to reverse terminally metabolically collapsed Tex cells.

The remodeling of the T cell community by the microenvironmental lipid network exhibits pronounced molecular-structure dependence and dynamic feedback characteristics. Ma et al. discovered that targeted inhibition of SOAT1 leads to selective accumulation of saturated fatty acids, which specifically recruit CD8+ T cells through NF-kappaB signaling activation, thereby producing significant synergy with anti-PD-1 therapy (82). This mechanism reveals a critical paradox: different lipid species (saturated fatty acids, cholesterol, polyunsaturated fatty acids) may respectively drive immune activation and dysfunction, requiring intervention strategies with single-molecule precision. Furthermore, Conche et al. revealed the intricate bidirectionality of ferroptosis-immune interactions: although GPX4-deficient tumor cells undergoing ferroptosis release CXCL10 to recruit CD8+ T cells, the system also compensatorily recruits myeloid-derived suppressor cells (MDSCs) to re-establish immune tolerance, indicating the presence of strong negative feedback regulation in cell death-triggered immune dialogue (83).

Beyond lipid metabolism, amino acid deprivation constitutes another dimension of passive metabolic checkpoint. The common feature of these two classical axes is that they do not actively transmit inhibitory signals but instead starve immune cells by depleting microenvironmental substrates: high SLC3A2 expression gives tumor cells a competitive advantage in lysine uptake (25); ZNF207-driven transcriptional upregulation of the IDO1-kynurenine-AhR pathway pushes CD8+ T cells toward exhaustion through severe tryptophan depletion (26). It should be emphasized, however, that computationally inferred metabolic checkpoints such as SLC3A2 require rigorous orthogonal validation before they can be considered actionable therapeutic targets. AI-based analysis of transcriptomic data can nominate SLC3A2-mediated lysine competition as a potential mechanism of T cell starvation, but transcript-level inference alone cannot establish the protein abundance, spatial distribution, or functional significance of this transporter. Quantitative multiplexed immunofluorescence imaging provides a critical next step by enabling statistical comparisons of SLC3A2 protein expression between distinct spatial compartments, specifically the tumor core and the invasive margin, while allowing direct assessment of whether transcript-inferred expression gradients are recapitulated at the protein level. Correlation analyses between imaging-derived protein abundance and sequencing-derived transcript abundance in matched tissue sections further quantify the reliability of computational predictions for this target. This validation framework, which progresses from transcriptomic inference to spatial protein measurement, is broadly applicable to AI-predicted metabolic checkpoints, including CD36, FASN, and SQLE. Powell and colleagues further demonstrated that a glutamine antagonist (JHU083) can potentiate PD-1 blockade through systemic metabolic reprogramming (84), although this has not been directly validated in HCC, suggesting that glutamine is also a critical dimension for maintaining T cell effector function. The immunological consequences of metabolic programs are often context-dependent on cell-type networks: Zhang et al. found that NOD1 activation in TAMs does not drive immunosuppressive FAO but instead, by suppressing FAO, leads to free fatty acid accumulation that paradoxically enhances CD8+ T cell activation via OX40L signaling (85), breaking the dogma that FAO in TAMs is invariably pro-tumorigenic. In the innate immune branch, iNKT cell functional direction is similarly governed by the microenvironmental metabolic spectrum: their reversal from pro-inflammatory to pro-tumor roles during HCC progression is tightly coupled to stage-specific evolution of lipid antigen repertoires (86) and acylcarnitine accumulation-induced cellular senescence (27).

### 5.2 Metabolic reprogramming and niche reconstruction of myeloid cells

In the HCC microenvironment, macrophages exhibit extraordinary state plasticity; their evolution from a pro-inflammatory to an immunosuppressive state is a continuum driven by the dynamic balance of metabolic fluxes, particularly glycolysis and FAO.

Spatial multi-omics and computational inference consistently demonstrate that M2-polarized TAMs are highly enriched in the metabolically active core zone at the tumor invasive front. In these spatial niches, TAMs not only rely on FABP5/FABP1-mediated FAO for metabolic fuel and sustained secretion of IL-10 and TGF-beta (15, 16), but also achieve persistent maintenance of their polarization state through the lactate-lactylation epigenetic axis. Lactate released by glycolysis in the HCC microenvironment drives H3K18 histone lactylation, which is not merely a transient metabolic sensor but establishes a powerful self-sustaining positive feedback network through SRSF10 and NUPR1, persistently anchoring macrophages to the pro-tumor M2 state (20, 21). In maintaining the stability of this state, Hao et al. further revealed that APOC1 safeguards M2 TAM survival by inhibiting ferroptosis-related gene expression, deeply coupling lipid metabolism, ferroptosis, and polarization reprogramming at the molecular level (87).

Based on these highly heterogeneous single-cell metabolic atlases, the communication networks through which myeloid cells act as central organizers of the TIME can be precisely reconstructed. Distinct macrophage subpopulations exploit specific metabolic-chemokine cascades to cooperatively construct immune barriers within their respective physical spaces: VIM-high macrophages at the tumor margin precisely recruit Tregs through IL-1beta (40); lipid droplet-laden macrophages chemotactically attract Tregs via the CCL20-CCR6 axis (13); and TAMs at the invasive margin specifically drive functional paralysis of MAIT cells (29). Even more refined is the identification by Yang et al., using integrative analysis, of a “ligand-receptor-metabolite” tripartite interaction between CD3+C1q+ double-positive TAMs and CCL4+ CD8+ T cells, confirming that metabolites themselves are high-level encoders of intercellular communication (88). This metabolically driven communication network even transcends the boundaries of conventional immune cells: Zhu et al. found that CD36+ CAFs can recruit CD33+ MDSCs through MIF secretion, constructing an unusual “CAF-MDSC-T cell” three-tier inhibitory cascade (89); Sun et al. extended this perspective to the circulatory system, demonstrating that circulating tumor cells also achieve systemic immune escape through CCL5-mediated Treg recruitment (90).

Finally, large-scale multi-omics subtyping reveals the profound etiology specificity and cross-tumor diversity of myeloid cell metabolic reprogramming. Zhou et al., through comparative analysis, confirmed that the myeloid composition of HCC differs significantly in spatial and functional terms from that of iCCA and combined hepatocellular-cholangiocarcinoma (91). Li et al. stratified HCC into three metabolic subtypes (F1-F3) based on fatty acid degradation pathways, finding that in MASLD-associated HCC, the spatial interaction topology of the MDSC-TAM-CD8+ T cell axis differs fundamentally from that in viral HCC (92). Prawira et al. identified a Treg-CAF-specific TNFSF14-TNFRSF14 axis in steatotic liver disease-driven HCC, further substantiating this argument (93). Additionally, the CCL4+ and PD-L1+ tumor-associated neutrophil subsets resolved by Xue et al. through single-cell omics (5) add new high-dimensional features to the HCC immune microenvironment; their distinctive metabolic behaviors, such as NETosis and aberrant lipid uptake, will undoubtedly become new focal points for precise AI-integrated multi-omics analysis.

## 6 Clinical translation: precision targeting of the immune-metabolic network

### 6.1 Next-generation combination therapy strategies

The metabolic checkpoint concept opens an entirely new translational dimension for HCC combination therapy strategies. Unlike conventional ICIs that block downstream signaling, metabolic checkpoint inhibitors aim to correct the metabolic etiology of immune dysfunction upstream; they are, in principle, mechanistically complementary to ICIs and capable of producing synergistic effects.

Anti-CD36 targeted therapy represents the most advanced metabolic checkpoint strategy in preclinical development. The first anti-CD36 IgG4 antibody (PLT012), developed by Tzeng et al., demonstrated TIME remodeling and antitumor activity in HCC xenograft and *in vitro* models (9); it remains, to our knowledge, the only HCC metabolic immune checkpoint-targeting antibody to have advanced to formal preclinical development, and no clinical trial in HCC patients has been initiated. However, the broad expression of CD36 in normal tissues such as adipose and cardiac muscle means that systemic blockade faces the severe challenge of on-target off-tumor toxicity. In the drug-repurposing pathway, the SQLE inhibitor terbinafine, an antifungal agent approved by the US Food and Drug Administration, restored anti-PD-1 efficacy in a MASH-HCC mouse model (12); whether the intrahepatic concentrations required for SQLE inhibition are achievable at standard antifungal dosing, and whether this efficacy extends to non-MASH etiologies of HCC, remain to be established. Additionally, FASN targeting (10), SOAT1 inhibition (82), and the pan-FAO inhibitor orlistat (15) have all demonstrated synergistic potential with anti-PD-1 in animal models. However, caution is warranted: metabolic side effects such as weight loss and hepatotoxicity induced by pan-FASN inhibition substantially limit the clinical advancement of this approach.

The lactate-lactylation epigenetic circuit offers multiple potential intervention nodes for the TIME, including inhibition of glycolytic kinases (e.g., HK2, PFKFB3), blockade of MCT4 efflux, inhibition of p300 (to block histone lactylation), and competitive antagonism using microbiota-derived D-lactate (23). Although MCT4 inhibitors have entered early-phase clinical trials in other cancer types, the feasibility of targeting the ACVR2A-MCT4-Treg axis in HCC awaits clinical data validation. In the hypoxia network, Myojin et al. found that an A2aR inhibitor reduces CD4+ T cell infiltration, providing theoretical support for combined targeting of the lactate and adenosine dual metabolic pathways (94).

In the adoptive cell therapy domain, metabolically engineered chimeric antigen receptor T (CAR-T) cells represent another frontier. Sun et al. demonstrated that overexpression of GLUT1 significantly enhances the glycolytic fitness and killing efficacy of CAR-T cells in the glucose-deprived HCC microenvironment (95). Even more elegantly, Hu et al. co-expressed AGK (acylglycerol kinase) and ADA1 (adenosine deaminase) in CAR-T cells, successfully converting the immunosuppressive adenosine in the microenvironment into a metabolic fuel directly utilizable by CAR-T cells (96). These proof-of-concept studies, conducted in HCC xenograft models, demonstrate that the metabolic fitness of CAR-T cells can be enhanced through ex vivo genetic engineering; however, long-term *in vivo* safety, target persistence, and manufacturing scalability remain unaddressed before clinical translation can be contemplated.

However, clinical or translational data from combining existing metabolic modulators with ICIs exhibit notable heterogeneity. In a MASH-HCC mouse model, Wabitsch et al. found that metformin restores CD8+ T cell function and potentiates anti-PD-1 efficacy (97), but no prospective trial has been conducted in HCC patients. Hu et al. also reported that interferon-alpha can potentiate anti-PD-1 in preclinical HCC models by remodeling glucose metabolism (98). In the high-profile LEAP-002 clinical trial (lenvatinib plus pembrolizumab), although the primary endpoint was not met, the lenvatinib monotherapy arm set a historic median OS record for HCC targeted therapy (19.0 months) (99). This inversely suggests that potent tyrosine kinase inhibitors may themselves have already profoundly influenced immune-metabolic dialogue through metabolic modulation and vascular normalization, thereby narrowing the therapeutic window for additional ICI benefit. Similarly, the ARTE trial of adding statins to the atezolizumab/bevacizumab backbone yielded negative results (100), potentially attributable to insufficient intratumoral drug concentrations or failure to effectively block HCC-specific cholesterol pathways. Collectively, these results point to a core clinical principle: metabolism-immune combination therapy cannot be applied broadly but must rely on exceptionally precise patient selection.

For different etiologies, metabolism-immune combination strategies must be differentially customized. For MASH/MASLD-associated HCC, intervention against PUFA-induced MAIT cell ferroptosis (28) or metformin-based restoration of CD8+ T cell function (97) holds potential targeting value. In viral HCC, blocking the acylcarnitine-iNKT cell senescence axis (27) or TIGIT-NECTIN2 interactions (34) may be more effective. Metabolic multi-omics subtyping studies have stratified HCC into three metabolic subtypes, providing a classification framework for etiology-oriented precision combination strategies (101). Yang et al. further subdivided HCC into four metabolic subtypes based on metabolic gene expression profiles and precisely identified palmitoyl-protein thioesterase 1 (PPT1) as a druggable target for specific subtypes (102), achieving a direct leap from metabolic classification to candidate drug nomination. Moreover, Xiong et al. used machine learning algorithms to extract a three-gene signature (CXCL8, SLC10A1, and ADH4) from lipid metabolism-related genes, providing a successful example of AI-assisted biomarker selection (103). The clinical translation progress and core bottlenecks of the major metabolic immune checkpoints and representative intervention strategies in the HCC microenvironment are summarized in **Table 2**.

**Table 2:** Clinical development status of major metabolic immune checkpoints.

Metabolic target/pathway	Representative drug/intervention	Current clinical/translational stage	HCC-specific efficacy evidence	Major challenges for clinical translation	Ref.
CD36 (Lipid metabolism)	PLT012 (humanized IgG4 antibody)	HCC preclinical only; no clinical trial initiated; non-human primate safety evaluation completed	Significant TIME remodeling in HCC xenograft/*in vitro* models	On-target off-tumor toxicity	(9)
SQLE (Cholesterol metabolism)	Terbinafine (antifungal drug repurposing)	FDA-approved for fungal infections (not for cancer); MASH-HCC mouse model only	Restores anti-PD-1 efficacy in MASH-HCC mouse model	Pharmacokinetic validation of effective intrahepatic concentration; etiology-specific limitations	(12)
FASN (Lipid synthesis)	Pan-FASN inhibitor	HCC preclinical only	Blocks Riplet-FASN axis-driven CD8+ T cell exhaustion	Severe systemic metabolic side effects (marked weight loss, hepatotoxicity)	(10)
MCT4 (Lactate efflux)	MCT4 small-molecule inhibitor	HCC preclinical only	ACVR2A-MCT4 axis promotes Treg recruitment in HCC animal models	Lack of real-world and prospective clinical data in HCC patient cohorts	(22)
GLS1 (Glutamine metabolism)	CB-839/JHU083	CB-839: Phase 1b/2 in non-HCC cancers (MDS, RCC, solid tumors). JHU083: preclinical DON prodrug only	Glutamine antagonism systemically potentiates anti-PD-1 therapy	Complex balance of glutamine uptake requirements between tumor and immune cells	(84)
A2aR (Adenosine metabolism)	A2aR receptor antagonist	Phase 1/2 clinical trials in non-HCC solid tumor immunotherapy; murine HCC models only	Reduces immunosuppressive T cell infiltration in murine HCC models	Limited HCC-specific mechanistic data; precise identification of responsive subsets needed	(94)
AMPK/mTOR (Energy metabolism)	Metformin/Rapamycin (repurposed)	FDA-approved for type 2 diabetes (not for cancer); MASH-HCC mouse model only	Metformin restores CD8+ T cell function in MASH-HCC model	Lack of prospective clinical trial evidence for HCC immunotherapy combination	(97)
Adoptive cell therapy (Metabolic engineering)	GLUT1/AGK/ADA1 overexpressing CAR-T	HCC preclinical proof-of-concept only; HCC xenograft models	Enhances killing efficacy in glucose-deprived and high-adenosine HCC microenvironment	Long-term *in vivo* safety, target persistence, and cell manufacturing complexity	(95, 96)

HCC, hepatocellular carcinoma; TIME, tumor immune microenvironment; MASH, metabolic dysfunction-associated steatohepatitis; FDA, US Food and Drug Administration; MDS, myelodysplastic syndromes; RCC, renal cell carcinoma; DON, 6-diazo-5-oxo-L-norleucine; AMPK, AMP-activated protein kinase; mTOR, mechanistic target of rapamycin; PD-1, programmed cell death protein 1; CAR-T, chimeric antigen receptor T-cell. Metabolic target abbreviations: SQLE, squalene epoxidase; FASN, fatty acid synthase; MCT4, monocarboxylate transporter 4; GLS1, glutaminase 1; A2aR, adenosine A2a receptor.

### 6.2 AI-assisted biomarker discovery

The primary prerequisite for precisely targeting the immune-metabolic network is the accurate identification of potential responder populations. AI models are demonstrating disruptive potential in this direction.

In the domain of pathological image analysis, Zeng et al. constructed a deep learning model capable of predicting HCC immune gene signatures directly from routine H&E-stained sections, achieving area under the curve values of 0.81-0.92 in retrospective analyses of HCC resection specimens (104). These findings suggest that tissue morphological features harbor high-dimensional information relevant to the microenvironmental immune state; however, the models were developed and tested on single-institution cohorts and have not undergone prospective validation. The same group further validated in a multicenter retrospective cohort the value of AI pathology scoring for predicting sensitivity to the atezolizumab-bevacizumab combination (105). At the imaging level, Wu et al. developed a magnetic resonance imaging-based radiomic immunoscore that, in a retrospective cohort, was associated with HCC immune microenvironment status and anti-PD-1 treatment response, effectively expanding the imaging modality for immune phenotyping from computed tomography to noninvasive magnetic resonance imaging (106).

In the dimension of liquid biopsy and dynamic monitoring, Matsumae et al. found that TERT promoter mutation status in circulating cell-free DNA, combined with serum alpha-fetoprotein levels, was associated with treatment outcomes of atezolizumab/bevacizumab in a retrospective cohort (107). At the metabolomics level, Pomyen et al. used serum metabolomic data from an Asian HCC cohort to define prognostic subtypes, supporting the feasibility of circulating metabolites as noninvasive biomarkers pending independent validation (108). Furthermore, Huo et al. constructed an 8-gene prognostic signature based on TAM metabolism-related genes, offering a molecular framework for prognostic stratification that awaits independent validation (109).

Nevertheless, it must be soberly recognized that the vast majority of current AI predictive models are trained on retrospective, single-center datasets and have not been validated in any prospective clinical trial. A decisive translational gap separates AI discovery from AI validation. Radiomics-based models are heavily dependent on hardware parameters and imaging settings of specific scanner types, with their cross-institutional generalizability remaining unclear, while transcriptomics-based AI models are chronically plagued by batch effects and cohort-specific biases.

### 6.3 Bridging the AI-clinical translation gap

The pathway from AI-driven metabolic checkpoint discovery to clinical application confronts a multifaceted translational gap that requires intermediate validation platforms and systematic characterization of discrepancies between computational predictions and clinical outcomes.

Patient-derived organoids (PDOs) provide a validation platform that is particularly well suited to HCC immunometabolism research because they preserve patient-specific tumor architecture and can be co-cultured with autologous immune cells to evaluate whether AI-predicted metabolic interventions produce the anticipated biological effects before advancing to *in vivo* studies. A recent study established a microfluidic PDO model from 30 patients with HCC in which tumor spheroids retained endogenous immune populations, including both lymphoid and myeloid cells, and exhibited immune-dependent responses to PD-1 blockade ex vivo, with 80% concordance relative to matched patient-derived xenografts. The authors further derived an Organoid Killing Index transcriptomic signature that correlated with clinical outcomes in patients treated with atezolizumab plus bevacizumab (R = 0.83, p < 0.001) (110). These findings demonstrate that PDO-based platforms can serve as a functional bridge between AI-nominated targets and clinical evaluation.

Microfluidic tumor-immune co-culture systems complement PDOs by recapitulating the spatial metabolic gradients, including oxygen tension, lactate concentration, and pH, that regulate immune cell behavior within the HCC microenvironment. Vascularized liver tumor-on-chip platforms have been shown to reproduce hypoxia-driven endothelial FasL upregulation and subsequent T cell apoptosis while enabling quantitative assessment of CAR-T cell cytotoxicity under controlled metabolic conditions (111). These systems allow investigators to isolate individual variables, such as lactate concentration or oxygen tension, while maintaining physiologically relevant co-culture architecture, thereby providing pharmacodynamic parameters that bridge the gap between static *in vitro* assays and whole-organism pharmacokinetics.

Beyond platform development, systematic characterization of the discrepancies between AI predictions and clinical immunotherapy responses must address four major challenges. First, spatial and temporal tumor heterogeneity means that a single biopsy may not represent the full spectrum of metabolic states within a tumor. Consequently, AI models trained on one region or time point may misclassify the metabolic phenotype of another. This limitation has been described as the “iceberg effect,” in which excellent internal validation metrics obscure poor external generalizability (112). Second, dynamic metabolic adaptation, whereby therapeutic inhibition of one metabolic checkpoint induces compensatory activation of alternative pathways, is not represented in pretreatment training datasets. Third, current training datasets underrepresent nonviral etiologies, including MASLD-associated HCC and alcohol-associated HCC, thereby limiting the generalizability of AI predictions across the full etiological spectrum of HCC. Fourth, transcriptome-inferred metabolic flux represents a computational surrogate rather than a direct measurement of enzymatic activity or metabolite abundance. The relationship between transcript abundance and real-time metabolic flux is further influenced by post-translational regulation, allosteric modulation, and substrate availability.

The broader challenge of biomarker generalizability in immuno-oncology is illustrated by the observation that PD-L1 immunohistochemistry, currently the most widely used clinical biomarker, was predictive in only 28.9% of US Food and Drug Administration approvals for immune checkpoint inhibitors (113). Addressing this translational gap will require integrating engineered human tissue platforms, spatially resolved longitudinal sampling, and paired transcriptomic and metabolomic datasets into the AI discovery and validation pipeline.

## 7 Conclusions and future perspectives

Three interlocking theoretical frameworks emerge from the evidence surveyed in this review: the metabolic checkpoint concept, the lactate-lactylation epigenetic axis, and the spatial metabolic gradient model. Synthesizing the current body of evidence, the CD36-governed “aberrant lipid uptake-ferroptosis” cascade emerges as the most mechanistically resolved metabolic immune checkpoint, with its specific blocking antibody already having entered preclinical translational development. Concurrently, the lactate-histone lactylation-M2 polarization axis profoundly reveals the epigenetic nature through which metabolites lock in immunosuppressive states via unique positive feedback mechanisms. At the spatial level, multiple independent spatial omics studies have consistently identified the tumor-stroma boundary, including the approximately 500 um invasive zone, as the critical physical interface of metabolic-immune interplay. Although current computational multi-omics frameworks have demonstrated remarkable capacity at the proof-of-concept stage, translating in silico predictions into experimentally confirmed biological mechanisms still requires systematic engineering advancement.

Examining the current landscape, several critical physical and algorithmic limitations that cannot be ignored constrain further progress in this field. The scarcity of paired omics data represents the primary bottleneck for current computational inference: existing AI tools that reverse-predict metabolic flux from mRNA abundance are fundamentally a mathematical surrogate strategy that cannot fully equate to actual metabolite concentrations, and emerging single-cell metabolomics mass spectrometry technologies have yet to achieve scalable application in complex HCC tissues. Furthermore, the causal validation loop in multi-omics research remains unclosed: AI algorithm-predicted metabolic master regulators generally lack systematic *in vivo* and *in vitro* causal validation, and verification models that combine CRISPR high-throughput screening with metabolic phenotype readouts remain absent from the HCC immune microenvironment. For multimodal data characterized by extreme HCC heterogeneity, systematic benchmarking of different computational integration methods is also conspicuously absent, leaving researchers without guidance for selecting optimal analytical practices. Moreover, the resolution of current mainstream spatial metabolomics (50–200 um) remains at the multi-cellular or micro-regional level, still unable to precisely decode the absolute metabolic state of individual immune cells.

Looking forward, HCC immuno-metabolism research harbors substantial breakthrough potential in the dimensions of multimodal fusion and clinical translation. With the explosion of generative AI, foundation models pretrained on over 33 million single cells, such as scGPT, have already demonstrated powerful generalization capacity (58); their deep fine-tuning on HCC-specific microenvironments is expected to achieve performance leaps in metabolic flux inference and virtual *in silico* perturbation. Concurrently, decoding the “microbiome-metabolite-immune” tripartite interaction space will become a major trend in reshaping the immune atlas. The liver, as the largest recipient of intestinal reflux blood, is profoundly influenced by microecological factors in its immune tolerance, yet only a handful of studies have ventured into this territory (23, 24). Future synchronous integration of HCC patients’ gut metagenomes, circulating metabolomes, and *in situ* single-cell immune atlases will substantially broaden the systemic understanding of liver organ-specific immune regulation. Moreover, exploration in this field should not be confined to classical glucose, lipid, and amino acid metabolism; dimensions such as one-carbon metabolism, nucleotide salvage pathways, and metallomics may well harbor next-generation metabolic immune checkpoints. The distinctive features of alcohol-associated liver disease-related HCC, such as direct immune cell destruction by acetaldehyde adducts and CYP2E1-induced oxidative stress, also urgently await systematic decoding. Ultimately, the definitive leap from computational discovery to clinical confirmation will critically depend on embedding AI-extracted multimodal biomarkers directly into umbrella or basket clinical trials of immune checkpoint inhibitors as core stratification factors.

Several outstanding questions will shape the next phase of this field. When will single-cell metabolomics achieve the throughput and resolution required for systematic validation of computationally inferred metabolic fluxes in HCC tissues? Which AI-predicted metabolic checkpoint, including CD36, FASN, SQLE, or a yet unidentified target, will be the first to enter a prospective clinical trial for patients with HCC? How can foundation models be effectively fine-tuned using HCC-specific multi-omics atlases, given the etiological diversity of the disease and the current underrepresentation of nonviral HCC in training datasets? Finally, will the gut microbiome-metabolite-immune axis reveal druggable vulnerabilities that can be therapeutically exploited to enhance responses to immunotherapy? Addressing these questions will require close integration of computational, experimental, and clinical research, an interdisciplinary effort that this review has sought to outline.

In summary, AI-driven single-cell multi-omics integration is reshaping HCC immunotherapy research into a paradigm that is mechanistic, predictive, and prescriptive. The formulation and validation of the metabolic checkpoint concept has fundamentally challenged the traditional view that metabolites are merely fuel for immune cells, recasting them as high-level encoders of immune cell states. The ultimate significance of this conceptual leap is that it opens a new druggable dimension for overcoming the HCC immunotherapy bottleneck. The successful translation of this dimension will determine whether the field of tumor immunometabolism can transcend the hype cycle of theoretical construction and truly mature into a historic breakthrough that extends survival for patients with HCC.
